# Comparative Osteogenesis of Radiopaque Dicalcium Silicate Cement and White-Colored Mineral Trioxide Aggregate in a Rabbit Femur Model

**DOI:** 10.3390/ma6125675

**Published:** 2013-12-05

**Authors:** Buor-Chang Wu, Shu-Ching Huang, Shinn-Jyh Ding

**Affiliations:** 1 School of Dentistry, Chung Shan Medical University, Taichung City 402, Taiwan; E-Mails: alanwu.adsl@msa.hinet.net (B.-C.W.); huang.mia@gmail.com (S.-C.H.); 2 Department of Dentistry, Chung Shan Medical University Hospital, Taichung City 402, Taiwan; 3 Institute of Oral Science, Chung Shan Medical University, Taichung City 402, Taiwan

**Keywords:** calcium silicate cement, root-end filling material, mineral trioxide aggregate, *in vivo*

## Abstract

The radiopaque dicalcium silicate cement (RDSC) displayed a shortened setting time and good biocompatibility. This study aimed to compare the regenerative potential of RDSC and white-colored mineral trioxide aggregate (WMTA) using a rabbit femur model. The animals were sacrificed at one, three and six months to accomplish histological and biochemical analyses. The results indicated that after one month of implantation, WMTA was associated with a greyish color alteration within its mass, while RDSC presented color stability even at six months. Histological assay with Masson’s Trichrome and Von Kossa stains showed the presence of newly formed bone surrounding the implanted sites in the rabbit femur. The histochemical data revealed that the RDSC group had significantly more bone regeneration than did the WMTA groups at three and six months. The conclusion drawn is that the encouraging results support the potential applications of RDSC as an improved alternative to WMTA for endodontic uses.

## 1. Introduction

Calcium silicate-based mineral trioxide aggregate (MTA) has been successfully used in endodontic treatment because of excellent biocompatibility, sealing ability and regenerative capabilities as well as antibacterial properties [1,2]. However, one of the main disadvantages of using MTA is its extended setting time and difficult handling [3,4]. Thus, efforts to develop novel endodontic materials as an alternative to MTA have been made, on the basis of overcoming its weakness [3,4,5]. In a previous study [5], we found that the aluminium-free hydraulic and radiopaque dicalcium silicate (β-Ca_2_SiO_4_) cement (RDSC) could set within 24 min when mixed with water, which the setting time was significantly lower than that of ProRoot white-colored mineral trioxide aggregate (WMTA) (168 min). The setting time is an important factor for satisfying clinical requirements, and a long setting time could cause problems clinically because of the cement’s inability to maintain its shape and support stresses within this time interval [6]. Faster setting times would allow thorough irrigation after root-end filling, minimizing debris left in the surgical site without risking material washout [7]. In addition, the dental pulp cell responses to RDSC are similar to those reported for WMTA in terms of cell cycle, proliferation, immunocompatibility and osteogenic differentiation, but the RDSC material has a better cell behavior [8]. More recently, it has been found that RDSC has high *in vitro* apatite-forming ability and low degradation [9]; thus, it may be an alternative to WMTA. Exposure of bioactive material surfaces such as MTA and calcium silicate to a physiological solution elicits the precipitation of a “bone-like” apatite layer, which may support the material’s ability to integrate into living tissue [10,11,12]. Gandolfi *et al.* [12] pointed out that calcium silicate cements showed a high calcium release and basifying effect, which resulted in a pronounced formation of more mature amorphous apatitic precursors. In addition, a synergistic effect between Si as an effective apatite nucleator and Ca as an apatite precipitation accelerator could cause an apatite precipitation [10].

*In vivo* models are required when *in vitro* systems cannot provide a reproducible approximation of the real-life *in vivo* or clinical setting [13]. The *in vivo* studies showed that MTA induces mineralized tissue formation such as dentin and cementum-like tissue [14,15,16]. Cintra *et al.* [15] found an MTA implant specimen showing irregular basophilic areas and hard tissues in close contact to the material after one-month implantation in alveolar bone of rats. MTA does not produce any adverse effect on the microcirculation of the connective tissue [17], but it could speed up the bone healing process [18]. Although abundant evidence in the literature has indicated that WMTA is effective for clinical applications, this field is continuously expanding. There have been few studies on *in vivo* osteogenesis of WMTA, given that the biomaterials should support bone ingrowth from the margins of the defects. In the present study, *in vivo* evaluation of RDSC was reported, in comparison with WMTA. To further clarify the differences in bone formation between the two cement specimens, the bone mineralization proteins such as osteocalcin (OC) and bone sialoprotein (BSP) during the bone formation were elucidated.

## 2. Experimental Section

### 2.1. Specimen Preparation

Reagent-grade tetraethyl orthosilicate (Si(OC_2_H_5_)_4_, Sigma-Aldrich, St. Louis, MO, USA) and calcium nitrate (Ca(NO_3_)_2_·4H_2_O, Showa, Tokyo, Japan) were used as precursors for SiO_2_ and CaO, respectively. The catalyst was 2 M nitric acid, and absolute ethanol was used as the solvent. The molar ratio of Ca(NO_3_)_2_·4H_2_O to Si(OC_2_H_5_)_4_ was 3:2. General sol-gel procedures, such as hydrolysis and aging, were adopted. A detailed description of the powder’s fabrication has been reported [19]. Briefly, Si(OC_2_H_5_)_4_ was hydrolyzed with the sequential addition of nitric acid and absolute ethanol with 1 h of stirring respectively. The required amount of Ca(NO_3_)_2_·4H_2_O was added to the above solution, and the mixed solutions were stirred for an additional hour. The sol solution was sealed and aged at 60 °C for 1 day. After vaporization of the solvent in an oven at 120 °C, the dried gel was heated in air to 800 °C at a heating rate of 10 °C/min for 2 h using a high-temperature furnace and then cooled to room temperature in the furnace to produce a powder. The sintered granules were then ball-milled for 12 h in ethyl alcohol using a centrifugal ball mill (Retsch S 100, Hann, Germany) and then dried in an oven at 60 °C. Bi_2_O_3_ (Sigma-Aldrich) (particle size 10 µm) was added to the ground powder at 20 wt % using a conditioning mixer (ARE-250, Thinky, Tokyo, Japan). To prepare the RDSC specimens the powder was hand-mixed with distilled water in a liquid-to-powder (L/P) ratio of 0.4 mL/g. ProRoot WMTA (Dentsply Tulsa Dental, Tulsa, OK, USA) was also regarded as an experimental group. The lot number and the expiration date of ProRoot WMTA were 09001921 and 04/2012, respectively. The liquid phase was water, and an L/P ratio of 0.3 mL/g was used according to the manufacturer’s instructions. After mixing, the cements were placed in a cylindrical Teflon mold to form cylindrical specimens with dimensions of 6 mm (diameter) × 2 mm (height); the specimens were stored in an incubator at 100% relative humidity and 37 °C for 1 day to set. Prior to implantation, the hardened cement discs were sterilized by soaking in a 75% ethanol solution and exposure to ultraviolet (UV) light for 2 h.

### 2.2. Surgery

**Figure 1:**
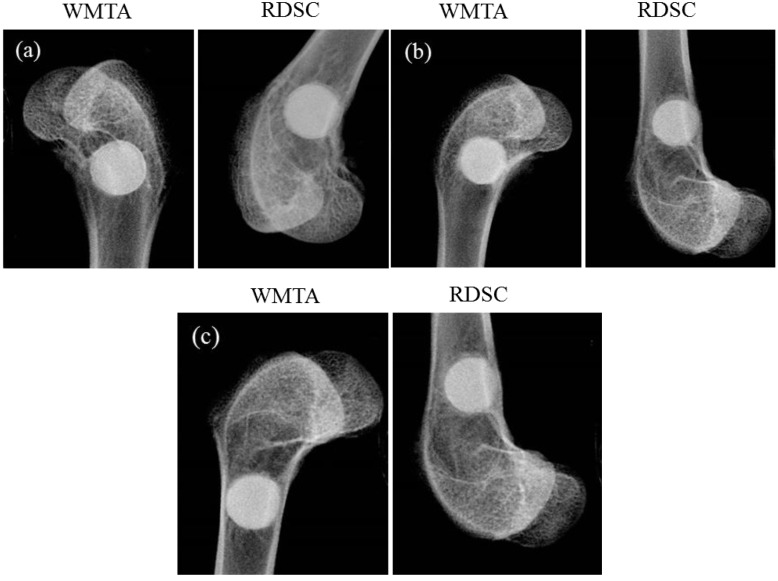
Radiographs of cements implanted in the defect of the distal medial femur for (**a**) 1; (**b**) 3; and (**c**) 6 months using a Minray dental X-ray unit (Soredex, Tuusula, Finland) operated at 60 kV and 7 mA with an exposure time of 0.1 sec. (white-colored mineral trioxide aggregate (WMTA); radiopaque dicalcium silicate cement (RDSC))

Fourteen male New Zealand white rabbits (4 months) with a weight of approximately 3 kg were used as experimental animals. The animal experiment was approved by the Animal Ethical Committee of the Chung Shan Medical University Experimental Animal Center and national guidelines for the care and use of laboratory animals were observed. Before surgery, the implant sites were shaved and sterilized with 70% ethanol 10% povidone iodine. Premedication of each animal was performed by intramuscular injecting Zoletil 50 (1 mL/kg) (Vibac Laboratories, Carros, France) and subcutaneous injecting atropine (0.5 mL/kg) (Septodont, St. Maur, France) for analgesia. For implantation of the cement samples, a 6 mm sized defect was created in the left and right distal medial femur using a low-speed trephine drill, as shown in Figure 1, and the incision was washed with normal saline in the process of defect preparation to eliminate bone debris. Each rabbit received two samples of test cement that could be put in the surgical defect securely without fixation. The bone defect without filling cement was used as a control. Following implantation of materials, the muscle and the skin were sutured using 4-0 polysorb (Syneture, Mansfield, MA, USA) in layers. Wounds were covered and protected with antibiotic ointment. The samples were harvested after 1, 3 and 6 months of implantation (three samples of each experimental group at each time point).

### 2.3. Color Stability

After sacrifice, implant areas were examined to observe the color stability of the cement samples under a light microscope (BH2-UMA; Olympus, Tokyo, Japan) equipped with a digital camera (C-5050; Olympus, Tokyo, Japan) at 50× magnification.

### 2.4. Histological Assay

The retrieved implants with their surrounding tissue were prepared for histological evaluation. The samples were fixed in 10% buffered formalin at 4 °C for 2 weeks and decalcified. Then the samples were embedded with paraffin. After which, 5 µm thick, longitudinal sections were prepared per specimen using a sawing microtome technique. The decalcified sections were prepared and stained with modified Masson’s Tricrome stain kit (ScyTek Lab., West Logan, UT, USA) and Von Kossa kit (ScyTek), according to the manufacturer’s instructions. Trichrome stain in blue was used for identification of collagen. Von Kossa staining in red can be used to observe the difference between the osteoid tissue and the calcified bone. Sections were examined using an Olympus BH2-UMA light microscope equipped with a digital camera (C-5050) at 200× magnification.

### 2.5. Western Blotting

Western blotting was used to evaluate the markers of osteogenesis on different cement specimens for a predetermined time to evaluate collagen type I (COL I), BSP, and OC levels. The region of interest (ROI) for Western blotting was 10 mm square around the cement using a dental bur before frozen in liquid nitrogen and protein isolation. The cement sample was removed by forceps and then frozen bone specimens were pulverized in a liquid-nitrogen-cooled vessel by pestle and mortar. Bone tissues were lysed in a 50 mM Tris buffer containing 1% Triton X-100 (Sigma, St. Louis, MO, USA), 20 μg/mL leupeptin (Sigma), and 0.5 mM phenylmethylsulfonyl fluoride (Sigma). The lysates were cleared by centrifugation at 15,000 × g. The protein concentrations of the lysates were measured using a Bio-Rad DC Protein Assay kit (Richmond, CA, USA), and proteins were resolved by standard sodium dodecyl sulfate-polyacrylamide gel electrophoresis (SDS-PAGE) and transferred to polyvinylidene difluoride (PVDF) membranes. After blocking with 5% BSA in PBS-T for 1 h, the membranes were incubated with primary antibodies against β-actin, COL I, BSP and OC (Abcam, Cambridge, MA, USA). A horseradish peroxidase (HRP)-conjugated secondary antibody was subsequently added, and the proteins were visualized by enhanced chemiluminescence. The stained bands were scanned and quantified using a densitometer (Syngene bioimaging system; Frederick, MD, USA) and Scion Image software (Frederick). Protein expression levels were normalized to the actin band for each sample.

### 2.6. Statistical Analysis

One-way analysis of variance (ANOVA) statistical analysis was used to evaluate the significance of differences between mean values. Scheffé multiple comparison testing was used to determine the significance of the deviations in the data for each specimen. In all cases, the results were considered statistically significant at a *p* value less than 0.05.

## 3. Results

### 3.1. Inflammatory Response

Bone tissue samples were excised from the site of implantation and analyzed using light microscopy. The macroscopic evaluation results showed that both cement implants and the control group exhibited no obvious inflammatory response, rejection or necrosis in the adjacent host tissue and they incorporated well with the surrounding tissue.

### 3.2. Degradation

The effects of implantation time on the degradation of two cement specimens are presented in Figure 1. Radiographs of cements implanted in the defect of the distal medial femur indicated the dimension of the cements did not change with time (Figure 1a,b). Even after six months of implantation the cement specimens underwent little change in diameter (Figure 1c), independent of the type of cement specimens. More importantly, it seems that the materials were in close contact to the bone tissue without the formation of fibrous interface.

### 3.3. Color Stability

**Figure 2:**
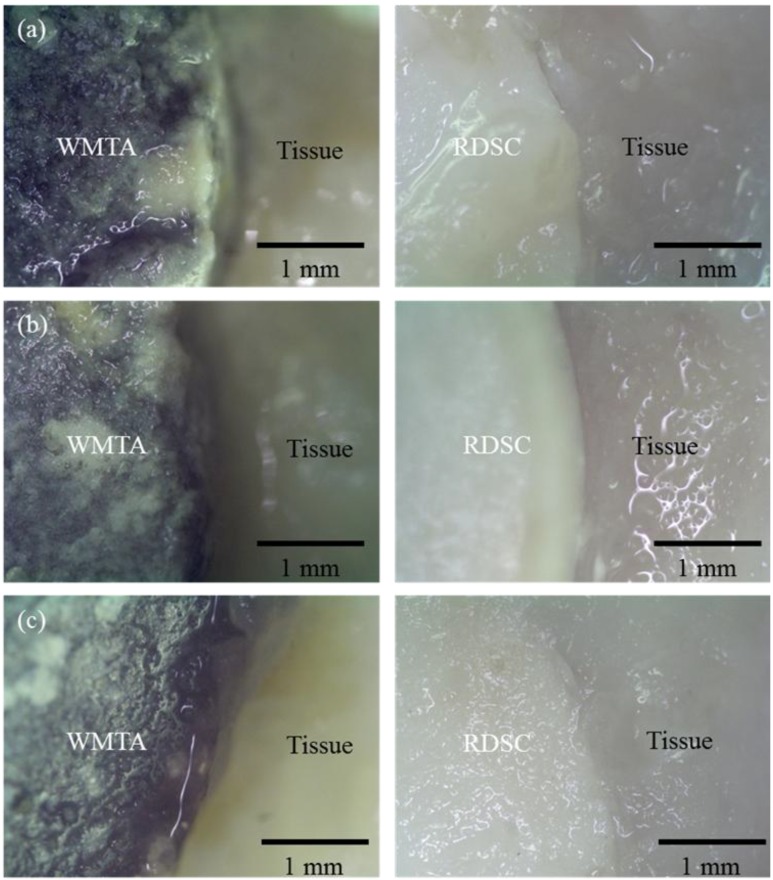
Photographs of WMTA and RDSC implants after (**a**) 1; (**b**); 3 and (**c**) 6 months of implantation. Of note, with WMTA there appeared a greyish discoloration at all implantation time-points. In contrast, RDSC maintained the original color. (Original magnification 50×).

Figure 2 shows color changes in the implanted materials after 1, 3 and 6 months of implantation. It is worthwhile to note that WMTA showed the greyish (or dark) discoloration at month 1, while RDSC implants kept the original color (Figure 1a). The RDSC group did not exhibit discoloration even after 3-month (Figure 2b) and 6-month implantation (Figure 2c).

### 3.4. Histological Observation

**Figure 3:**
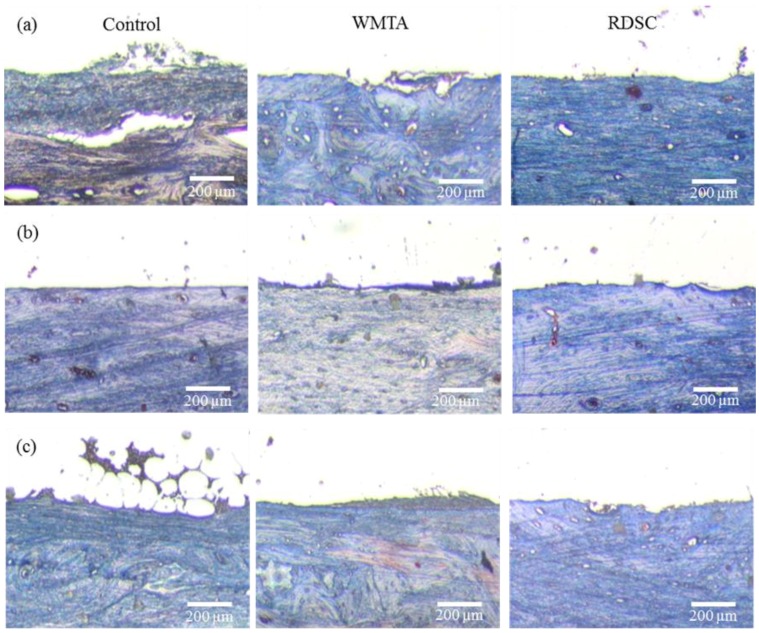
Modified Masson’s Trichrome staining photographs of rabbit femur surrounding the control, WMTA, and RDSC for different implantation time-points. (**a**) The 1-month WMTA and RDSC implant samples showed the deposition of collagen surrounding the implanted sites; (**b**) More staining was seen at the bony tissue adjacent to implant; (**c**) After six months implantation sites were surrounded by a connective tissue that was organized and dense with the presence of osteoblasts and osteoid matrix for all groups. (Original magnification 200×).

The surrounding tissue of the retrieved implants was analyzed after 1, 3 and 6 months of implantation. The control group, at month-1 of observation, showed collagen formation (Figure 3a). Similarly, the two cements samples stained with Masson’s Trichrome at month-1 showed deposition of collagen surrounding the implanted sites which increased quickly over time. This process was characterized by a great quantity of connective tissue surrounding the bone defect area, which might elicit one of the initial phases of the bone healing process (Figure 3b). The implantation site was surrounded by connective tissue that was organized and dense with the presence of osteoblasts and osteoid matrix (Figure 3c). The staining in WMTA and RDSC groups showed no significant differences at all implantation time-points.

**Figure 4:**
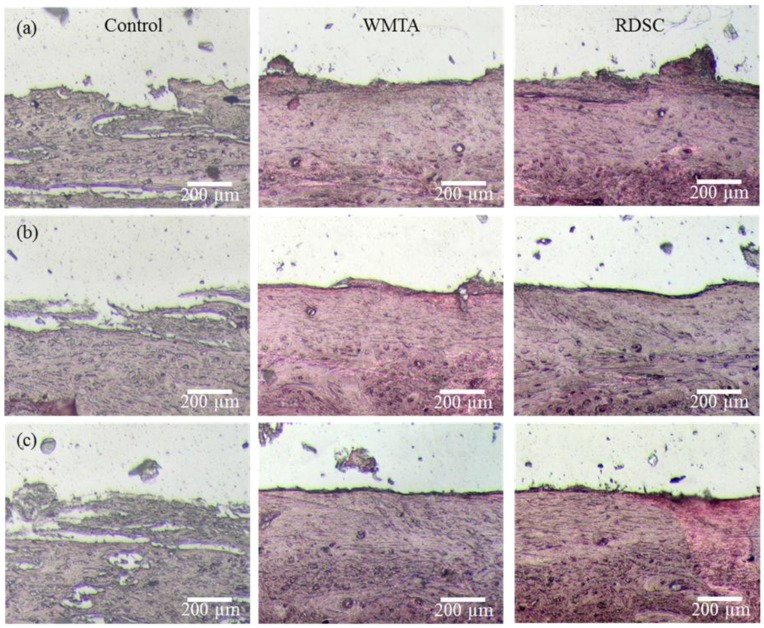
Von Kossa staining photographs of rabbit femur surrounding the control, WMTA, and RDSC for different implantation time-points. (**a**) At 1-month the control was only lightly stained. WMTA and RDSC began to deposit mineral and the degree of bone apposition; (**b**) Mineral deposition in large areas was also confirmed at 3-month implantation; (**c**) Six months after implantation, osteoid formation was appreciated in the two cements at the defect edges. (Original magnification 200×).

Mineralization was localized around the implants and displayed by black mineral in tissue samples stained with Von Kossa. Black, red, and pink correspond to calcified areas, nuclei, and cytoplasm, respectively. After one month of implantation, the control was only lightly stained (Figure 4a). The images of sections stained showed that WMTA and RDSC began to deposit mineral and the degree of bone apposition was better than the control group and exhibited a superficial layer with basophilic areas. Newly grown bone tissue in direct contact with cement surfaces was clearly visible one month after surgery. Mineral deposition in large areas was also confirmed by Von Kossa assay harvested *in vivo* at three (Figure 4b) and six months (Figure 4c) after implantation. Six months after implantation, osteoid formation was appreciated in the two cements at the defect edges.

### 3.5. Bone-Related Protein Expression

**Figure 5:**
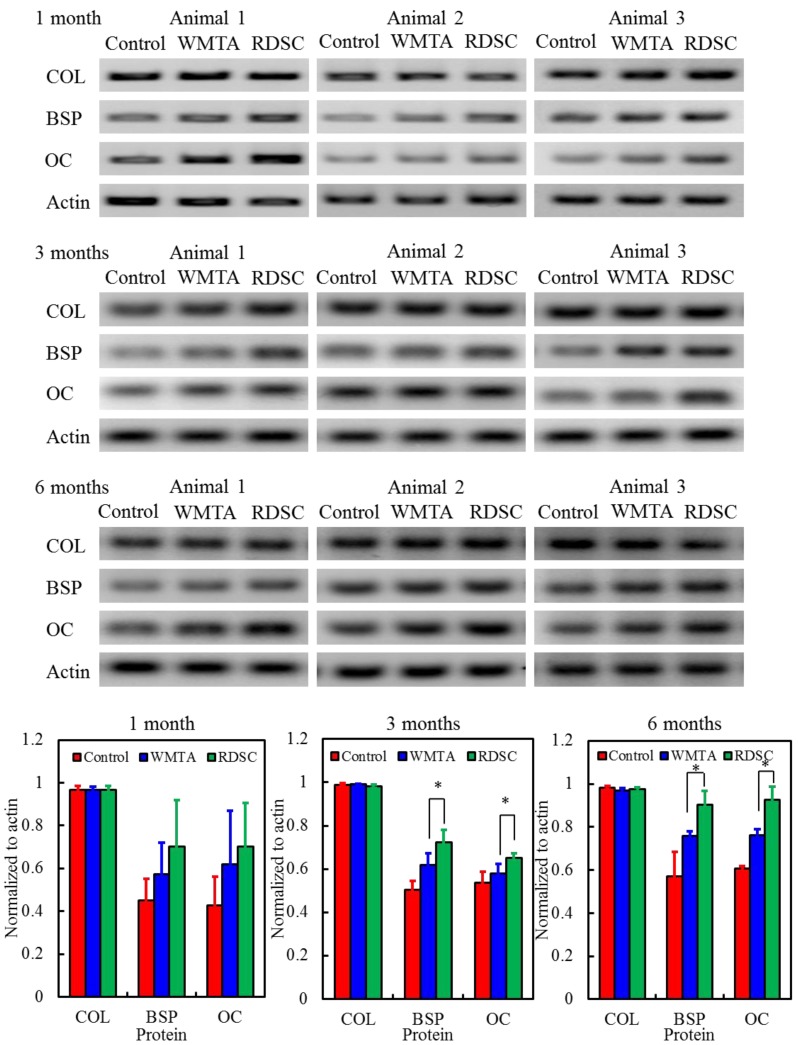
The bone-related protein expression of collagen type (COL), bone sialoprotein (BSP), and osteocalcin (OC) levels normalized to actin for bone tissue by Western blotting after 1, 3, and 6 months of implantation. It can be clearly seen that BSP and OC levels were higher in cells surrounding the RDSC cement specimens than in cells for the WMTA after three and six months.

Changes in the protein expression of cells surrounding the implantation sites with varying culture durations are shown in Figure 5. Actin was used as an internal control and was produced at a comparable level in the control group and cement groups. The blotting patterns of COL I protein were also similar in the control and cement groups during the implantation time periods. The quantitative analysis indicated no significant difference (*p* > 0.05) in COL I levels between all test groups. In contrast, BSP and OC levels were higher in cells surrounding the two cement specimens than in cells for the control. Although no significant differences (*p >* 0.05) in the two bone-related protein levels were detected between the two cements at month-1, the results of 3 or 6 month-implantation presented significantly different (*p <* 0.05). For example, at month-3, a significant 17% increment in BSP level was measured for the RDSC group compared to the WMTA group and the increment became 19% at six months of implantation.

## 4. Discussion

### 4.1. Inflammatory Response

The *in vivo* assay was tested to further contribute to research in this area and evaluate the potential for use in clinical trials. The two cement groups showed normal bone development with no inflammation during the implantation time. It is important to highlight the absence of inflammatory cells or acute inflammation processes at the interfaces between tissue and implanted material, in agreement with previous studies [7,20,21]. McNamara *et al.* [7] reported that grey-colored MTA displays no inflammation at 8-week implantation into rat mandibles. In a dog’s teeth model grey-colored MTA was associated with less periapical inflammation and tissue response, even when no root filling or coronal restoration was present [20].

### 4.2. Degradation

An important requirement for any dental material used for permanent treatment is its resistance to degradation (or solubility) when exposed to a host environment for a prolonged time period. The two cement samples exhibited few variations in size even after six months of implantation (Figure 1c). WMTA is used as root-end filling materials that are in direct contact with the periapical tissues and for this reason, the ideal material should be biocompatible, impervious to dissolution or breakdown by the tissue fluids, nonresorbable, adapting as closely as possible to the dentinal walls of the root-end preparation and possess good handling characteristics. Shie *et al.* [22] found that WMTA was associated with a relatively small degree of weight loss even after a 7-day soaking period in a pH 4.0 physiologic solution. Gandolfi *et al.* [5] also reported the increase of the weight of calcium silicate-based materials when they came in contact with simulated body fluids. As previously stated, high apatite forming activity and low degradation were the characteristics of RDSC [9].

### 4.3. Color Stability

In addition to the noted shortcomings in setting time, the potential of discoloration of WMTA has been a concern. Indeed, severe greyish (or dark) discoloration of WMTA was found in the present *in vivo* results (Figure 2). In contrast, RDSC presented color stability. The unexpected findings have also been reported in *in vitro* and *ex vivo* studies related to the color stability of WMTA [23,24,25,26,27]. Although the exact explanation for WMTA-induced discoloration is not yet understood, the difference in chemical composition between the two cements can be one factor. WMTA is typically comprised of a variety of oxide components such as SiO_2_, CaO, and Al_2_O_3_ with trace FeO [26], while RDSC consists of SiO_2_ and CaO. Felman and Parashos suggested that the oxidation and incorporation of the remaining iron content within the WMTA powder into the calcium aluminoferrite phase of the set WMTA in the presence of blood might be the mechanism of discoloration [26].

### 4.4. Histological Morphology

Masson’s trichrome was used to stain the harvested tissue to visualize the formation of collagen in blue. The collagen fibers may indicate matrix development for future mineralization and bone formation (Figure 3). Mineralization borders were also identified with Von Kossa staining. Histological evaluation revealed that the two cement implants were encapsulated by the surrounding bone tissue, and the new bone was in direct contact with the implant at 1-month after implantation (Figure 4). As an ideal implanted biomaterial candidate, the cement samples studied herein presented good biocompatibility and osteoconductive properties. The significant findings, from a clinical perspective, were the osteogenesis with which both cements can induce new bone formation.

The implantation of both RDSC and WMTA samples into bone defects in rabbit femur resulted in enhanced mineral deposition *in vivo*. The results may be partially explained by the fact that a similarity in chemical composition and biocompatibility occurred [8]. According to the literature [28,29], calcium silicate-based cements could remineralize the partially demineralized dentin and induce calcium-phosphate deposit formation. Dreger *et al.* [30] reported that calcium silicate-based MTA or Portland cements released some of their components in the tissue capable of stimulating mineral deposition in the cement-dentin interface and in the interior of the dentinal tubules. When a biomaterial is implanted into a body, a calcium and phosphate-rich layer forms on its surface, which then bonds to the living bone through this biologically active carbonated apatite layer [12]. On the other hand, that the presence of calcium carbonate (calcite) can be confirmed on the surface of calcium silicate cement when in contact with simulated body fluids [31]. Bortoluzzi *et al.* [32] suggested that WMTA could lead to formation of calcite granules and underlying bridges of mineralized tissue because of the release of calcium ions that react with the carbonate of the tissues. Xu *et al.* [33] found that newly formed bone tissue grew into the porous calcium silicate-based materials in a rabbit calvarial defect model, along with the deposition of a bone-like apatite layer at the tissue/material interface. Nevertheless, it seems that the mineral deposition in the regenerated tissues was more extensive in the RDSC group than in the WMTA group at three and six months after implantation.

### 4.5. Bone-Related Protein

When the biomaterials are introduced into a bone defect, the binding initially occurs between the implanted samples and the bone tissue. Various growth factors and mesenchymal cells reside in the sample surfaces or surrounding the implants [6]. The quantitative experimental findings were confirmed after Western blotting assessment of ROI. Osteoblast differentiation is generally accompanied by OC and BSP as well as *in vitro* mineralization. A synchronized sequence of proteins must be activated in the osteoblasts surrounding the cements so that they undergo cell division and then synthesize an extracellular matrix that is capable of mineralizing to become bone. Collagen is the major constituent of organic bone matrix. In this study there were no significant differences in collagen expression among the three groups at all implantation time intervals. OC is a later marker of osteoblast differentiation and is the most abundant non-collagenous bone-matrix protein characteristic of osteoblast synthetic function [10,34]. Its expression increases rapidly as mineralization increases. BSP is specific to mineralized tissues and is expressed at the onset of bone formation during embryogenesis [34]. The two bone-related proteins, OC and BSP, exhibited different blotting patterns of expression from collagen. We did observe noticeable differences in the levels of BSP and OC between WMTA and RDSC. A quantitative analysis showed that the bone-specific protein values (BSP and OC) for the RDSC specimens were significantly higher than the values for WMTA, which indicated that more effective osteogenesis and oseointegration had occurred around the bone defect area (Figure 5). Compared to WMTA, the enhanced bone formation on RDSC might result from the direct contact of migrating osteogenic cells from the surrounding tissues, which then stimulates cell proliferation and osteogenic differentiation. In an earlier study [4], we reported that RDSC could possess significantly more pronounced cell proliferation than WMTA after culture in MG63 cells for seven days.

### 4.6. Material Comparison

The chemical composition of implant materials has been shown to affect cell behavior, including cell shape, attachment, proliferation, differentiation and mineralized matrix synthesis [35,36,37]. Ion-releasing calcium silicate cements support a biomimetic microenvironment conducive to survival and differentiation of human orofacial bone mesenchymal stem cells [38]. The previous investigation performed with cementoblast cells demonstrates the ability of WMTA to allow cell growth and to induce biomineralization [14]. In this study, the major difference in the levels of BSP and OC appears to be the Ca/Si ratio of the two calcium silicate cements. The Ca/Si atomic ratio of WMTA is more than 3 [39,40], which indicates higher than RDSC of 1.5. It seems therefore reasonable to suspect that RDSC has a higher Si content in the materials’ mass than WMTA. Bioactive Si-containing substrates, which release soluble silicate species, have been shown to accelerate the formation of new bone tissue by promoting the genetic activity of bone-regulating cells [36]. An appropriate Si concentration is effective in supporting the proliferation of osteoblast-like cells and actively stimulating a biological response in MG63 cells through the production of bone-specific proteins [41]. More importantly, the cement with a higher Si content is beneficial for collagen adsorption and secretion, in which in turn more cells remains on the cement surfaces with higher Si content than on those with lower Si content [35]. Previous *in vitro* studies in our laboratory documented that ALP activity and bone-related gene expression such as OC and BSP in MG63 cells increased significantly (*p* < 0.05) with an increase in the Si content of calcium silicate cements [36]. It is reasonable to speculate that RDSC has the better expression patterns of bone-related proteins in an *in vivo* system when compared to WMTA. Last but not least, the RDSC provided a favorable environment for bone tissue ingrowth, resulting in a large amount of mineralized tissue formation.

## 5. Conclusions

RDSC implants were implanted in the rabbit femur defects to evaluate the *in vivo* bone-regenerative capacity of RDSC, and the results were compared to WMTA implants. Contrary to RDSC having color stability, noted discoloration of WMTA was found. Histological assay with Masson’s Trichrome and Von Kossa staining showed abundant osteoid and mineralized bone tissue between the cement-tissue interfaces compared with the control without implants. Quantitative analysis results confirmed that the RDSC implants exhibited more effective osteogenesis surrounding the bone defect area than with WMTA implants. The encouraging results support the potential applications of RDSC as an improved alternative to WMTA for endodontic uses.

## References

[1] Parirokh M., Torabinejad M. (2010). Mineral trioxide aggregate: A comprehensive literature review—Part I: Chemical, physical, and antibacterial properties. J. Endod..

[2] Torabinejad M., Parirokh M. (2010). Mineral trioxide aggregate: A comprehensive literature review—Part II: Leakage and biocompatibility investigations. J. Endod..

[3] Camilleri J., Montesin F.E., Di Silvio L., Pitt Ford T.R. (2005). The chemical constitution and biocompatibility of accelerated Portland cement for endodontic use. Int. Endod. J..

[4] Chiang T.Y., Ding S.J. (2010). Comparative physicochemical and biocompatible properties of radiopaque dicalcium silicate cement and mineral trioxide aggregate. J. Endod..

[5] Gandolfi M.G., Taddei P., Siboni F., Modena E., Ciapetti G., Prati C. (2011). Development of the foremost light-curable calcium-silicate MTA cement as root-end in oral surgery. Chemical-physical properties, bioactivity and biological behaviour. Dent. Mater..

[6] Sugawara A., Asaoka K., Ding S.J. (2013). Calcium phosphate-based cements: Clinical needs and recent progress. J. Mater. Chem. B.

[7] McNamara R.P., Henry M.A., Schindler W.G., Hargreaves K.M. (2010). Biocompatibility of accelerated mineral trioxide aggregate in a rat model. J. Endod..

[8] Chen C.C., Shie M.Y., Ding S.J. (2011). Human dental pulp cells responses to new calcium silicate-based endodontic materials. Int. Endod. J..

[9] Chiang T.Y., Ding S.J. (2013). Physicochemical properties of radiopaque dicalcium silicate cement as a root-end filling material in an acidic environment. Int. Endod. J..

[10] Ding S.J., Shie M.Y., Wang C.Y. (2009). Novel fast-setting calcium silicate bone cements with high bioactivity and enhanced osteogenesis *in vitro*. J. Mater. Chem..

[11] Gandolfi M.G., Taddei P., Tinti A., Prati C. (2010). Apatite-forming ability of ProRoot MTA. Int. Endod. J..

[12] Gandolfi M.G., Taddei P., Modena E., Siboni F., Prati C. (2013). Biointeractivity-related versus chemi/physisorption-related apatite precursor-forming ability of current root end filling materials. J. Biomed. Mater. Res..

[13] Muschler G.F., Raut V.P., Patterson T.E., Wenke J.C., Hollinger J.O. (2010). The design and use of animal models for translational research in bone tissue engineering and regenerative medicine. Tissue Eng. B.

[14] Hakki S.S., Bozkurt S.B., Hakki E.E., Belli S. (2009). Effects of mineral trioxide aggregate on cell survival, gene expression associated with mineralized tissues, and biomineralization of cementoblasts. J. Endod..

[15] Cintra L.T.A., de Moraes I.G., Estrada B.P.F., Gomes-Filho J.E., Bramante C.M., Garcia B., Bernardinelli N. (2006). Evaluation of the tissue response to MTA and MBPC: Microscopic analysis of implants in alveolar bone of rats. J. Endod..

[16] Bernabé P.F.E., Holland R., Morandi R., de Souz A.V., Nery M.J., Otoboni-Filho J.A., Dezan Junior E., Gomes-Filho J.E. (2005). Comparative study of MTA and other materials in retrofilling of pulpless dogs’ teeth. Braz. Dent. J..

[17] Masuda Y.M., Wang X., Hossain M., Unno A., Jayawardena J.A., Sato K., Nakamura Y., Matsumoto K. (2005). Evaluation of biocompatibility of mineral trioxide aggregate with an improved rabbit ear chamber. J. Oral Rehabil..

[18] do Nascimento C., Issa J.P.M., Iyomasa M.M., Regalo S.C.H., Siéssere S., Pitol D.L., de Oliveira Wolga N., Pedrazzi V. (2008). Bone repair using mineral trioxide aggregate combined to a material carrier, associated or not with calcium hydroxide in bone defects. Micron.

[19] Chiang T.Y., Wei C.K., Ding S.J. Effects of bismuth oxide on physicochemical properties and osteogenic activity of dicalcium silicate cements. J. Med. Biol. Eng..

[20] Bernabé P.F.E., Gomes-Filho J.E., Rocha W.C., Nery M.J., Otoboni-Filho J.A., Dezan-Júnior E. (2007). Histological evaluation of MTA as a root-end filling material. Int. Endod. J..

[21] Parirokh M., Mirsoltani B., Raoof M., Tabrizchi H., Haghdoost A.A. (2011). Comparative study of subcutaneous tissue responses to a novel root-end filling material and white and grey mineral trioxide aggregate. Int. Endod. J..

[22] Shie M.Y., Huang T.H., Kao C.T., Huang C.H., Ding S.J. (2009). The effect of a physiological solution pH on properties of white mineral trioxide aggregate. J. Endod..

[23] Jacobovitz M., de Lima R.K. (2008). Treatment of inflammatory internal root resorption with mineral trioxide aggregate: A case report. Int. Endod. J..

[24] Belobrov I., Parashos P. (2011). Treatment of tooth discoloration after the use of white mineral trioxide aggregate. J. Endod..

[25] Boutsioukis C., Noula G., Lambrianidis T. (2008). *Ex vivo* study of the efficiency of two techniques for the removal of mineral trioxide aggregate used as a root canal filling material. J. Endod..

[26] Felman D., Parashos P. (2013). Coronal tooth discoloration and white mineral trioxide aggregate. J. Endod..

[27] Ioannidis K., Mistakidis I., Beltes P., Karagiannis V. (2013). Spectrophotometric analysis of coronal discolouration induced by grey and white MTA. Int. Endod. J..

[28] Tay F.R., Pashley D.H. (2008). Guided tissue remineralisation of partially demineralised human dentine. Biomaterials.

[29] Gandolfi M.G., Taddei P., Siboni S., Modena E., de Stefano E.E., Prati C. (2011). Biomimetic remineralization of human dentine using promising innovative calcium-silicate hybrid “smart” materials. Dent. Mater..

[30] Dreger L.A.S., Felippe W.T., Reyes-Carmona J.F., Felippe G.S., Bortoluzzi E.A., Felippe M.C.S. (2012). Mineral trioxide aggregate and Portland cement promote biomineralization *in vivo*. J. Endod..

[31] Gandolfi M.G., Taddei P., Tinti A., Dorigo De Stefano E., Rossi P.L., Prati C. (2010). Kinetics of apatite formation on a calcium-silicate cement for root-end filling during ageing in physiological-like phosphate solutions. Clin. Oral Invest..

[32] Bortoluzzi E.A., Broon N.J., Bramante C.M., Consolaro A., Garcia R.B., de Moraes I.G., Bernadineli N. (2008). Mineral trioxide aggregate with or without calcium chloride in pulpotomy. J. Endod..

[33] Xu S., Lin K., Wang Z., Chang J., Wang L., Lu J., Ning C. (2008). Reconstruction of calvarial defect of rabbits using porous calcium silicate bioactive ceramics. Biomaterials.

[34] Hakki S.S., Bozkurt B.S., Gandolfi M.G., Prati C., Belli S. (2013). The response of cementoblasts to calcium phosphate resin-based and calcium silicate-based commercial sealers. Int. Endod. J..

[35] Shie M.Y., Ding S.J. (2013). Integrin binding and MAPK signal pathways in cell responses to surface chemistry of calcium silicate cements. Biomaterials.

[36] Shie M.Y., Chang H.C., Ding S.J. (2012). Effects of altering the Si/Ca molar ratio of a calcium silicate cement on in vitro cell attachment. Int. Endod. J..

[37] Gandolfi M.G., Ciapetti G., Taddei P., Perut F., Tinti A., Cardoso M., VanMeerbek B., Prati C. (2010). Apatite formation on bioactive calcium-silicate cements for dentistry affects surface topography and human marrow stromal cells proliferation. Dent Mater..

[38] Gandolfi M.G., Shah S.N., Feng R., Prati C., Akintoye S.O. (2011). Biomimetic calcium-silicate cements support differentiation of human orofacial bone marrow stromal cells. J. Endod..

[39] Asgary S., Parirokh M., Eghbal M.J., Brink F. (2005). Chemical differences between white and gray mineral trioxide aggregate. J. Endod..

[40] Dammaschke T., Gerth H.U.V., Zűchner H., Schäfer E. (2005). Chemical and physical surface and bulk material characterization of white ProRoot MTA and two Portland cements. Dent. Mater..

[41] Shie M.Y., Ding S.J., Chang H.C. (2011). The role of silicon in cell proliferation and apoptosis. Acta Biomater..

